# A cognitive model of pathological worry

**DOI:** 10.1016/j.brat.2012.06.007

**Published:** 2012-10

**Authors:** Colette R. Hirsch, Andrew Mathews

**Affiliations:** a King's College London, UK; b University of Western Australia, Australia; c University of California, Davis, USA

**Keywords:** Worry, Emotional processing biases, Attention, Interpretation, Attentional control

## Abstract

We present an evidence-based model of pathological worry in which worry arises from an interaction between involuntary (bottom-up) processes, such as habitual biases in attention and interpretation favouring threat content, and voluntary (top-down) processes, such as attentional control. At a pre-conscious level, these processes influence the competition between mental representations when some correspond to the intended focus of attention and others to threat distracters. Processing biases influence the probability of threat representations initially intruding into awareness as negative thoughts. Worry in predominantly verbal form then develops, influenced by conscious processes such as attempts to resolve the perceived threat and the redirection of attentional control resources to worry content, as well as the continuing influence of habitual processing biases. After describing this model, we present evidence for each component process and for their causal role in pathological worry, together with implications for new directions in the treatment of pathological worry.

## Highlights

► We describe an evidence-based cognitive model of pathological worry. ► Factors involved include processing biases, misdirected control and verbal encoding. ► Evidence shows that pathological worry is reduced by modifying processing biases. ► We predict that optimal results will depend on addressing each factor involved.

Worry is a primary cognitive characteristic of anxiety, and has been described as ‘a chain of thoughts and images, negatively affect-laden and relatively uncontrollable’ (Borkovec, Robinson, Pruzinsky, & DePree, 1983, p. 10). The content of worry typically concerns future events whose outcomes are uncertain, but contain the possibility of one or more negative outcomes (Sibrava & Borkovec, 2006). Reported proneness to worry varies continuously across the normal population, without any sudden discontinuity (Ruscio, Borkovec, & Ruscio, 2001). However, worry features prominently in emotional disorders, particularly Generalized Anxiety Disorder (GAD) in which uncontrollable worry about many different topics constitutes the main diagnostic criterion. In other disorders worry tends to be focused on more specific events, such as the anticipation of social embarrassment in social phobia. For this reason we will focus particularly on worry in GAD as the clearest form of pathological worry (i.e. it is general, excessive, uncontrollable and distressing), while recognizing that similar processes occur in other disorders.

Pathological worry has much in common with what has been described as rumination, more often studied in the context of depression (Nolen-Hoeksema, 1991). Both involve repetitive thinking about negative self-relevant topics, typically in quasi-verbal and rather abstract form, such as “What if something terrible happens?” (in worry); or “Why am I such a failure?” (in rumination). Factor analyses of questionnaires purporting to assess rumination or worry have not revealed clearly independent underlying factors (Segerstrom, Tsao, Alden, & Craske, 2000), so the two concepts may overlap, at least to some degree. However, the content typically labelled as worry concerns thoughts of possible future threats impinging on the individual, whereas the term rumination is more often applied to thoughts of past negative events or negative personal attributes (Watkins, Moulds, & Mackintosh, 2005). In any event, our primary concern here is to present a theoretical account of pathological worry, without making claims about the extent to which it may also apply to rumination.

Although anticipation of probable danger is adaptive, it is unclear why excessive worry about low probability events persists when it causes frequent mental distress, with so little apparent benefit. Borkovec and colleagues have long argued that worry is negatively reinforced by the avoidance of the greater emotional reactions associated with processing threats in the form of imagery (e.g. Borkovec, Alcaine, & Behar, 2004). In a modified version of the avoidance model, Newman and Llera (2011) present evidence that, rather than avoiding imagery, worry is maintained by the perception that it serves to avoid further increases in distress should a feared event actually occur. Several research groups (e.g., Davey, 2006; Ruscio & Borkovec, 2004; Wells, 1995) have noted that excessive worriers sometimes endorse certain advantages for worry (e.g. that worry helps them to solve problems, or allows dangers to be avoided), despite also endorsing negative beliefs (e.g., that worry is uncontrollable or may be harmful to health), and have suggested that these beliefs promote worry. Other researchers have further suggested that individuals who worry excessively are intolerant of uncertainty and believe that they must continue to worry until uncertainty has been resolved (Dugas, Gagnon, Ladouceur, & Freeston, 1998).

Aspects of the model to be presented here overlap with previous accounts in which pathological worry (or GAD) is promoted by maladaptive beliefs (Wells, 1995), intolerance of uncertainty (Dugas et al., 1998) and inability to effectively regulate emotion (Mennin, Heimberg, Turk, & Fresco, 2005). Several of these factors may combine to promote worry, as suggested in the model offered by Berenbaum (2011), although evidence that they are causal – rather than being correlates or consequences of worry – is inconclusive. In the present model we focus on component processes for which there is evidence that they have a causal role in worry, and that lead to implications for treatment.

## Overview of the model

The building blocks in the proposed model are cognitive characteristics associated with pathological worry: these include biases in the processing of emotional information; depleted or misdirected executive control of attention, and the quasi-verbal form of worry itself. Note that we are *not* claiming that any one of these characteristics is *unique* to pathological worry, or one specific disorder; although we do argue that they combine in particularly potent form in pathological worry, and most obviously in GAD. The basic form of the model is first briefly outlined below; and supporting evidence for each component is then reviewed in the following sections, before finally turning to implications for testing its validity and for new treatment approaches.

### Emotional processing biases

High levels of anxiety and worry (e.g. in GAD) are characterized by selective attention to threatening cues matching emotional concerns (Mathews & MacLeod, 2005), or internally generated representations, such as bodily sensations, mental images or worrisome thoughts (Hayes, Hirsch, & Mathews, 2010; Krebs, Hirsch, & Mathews, 2010; Mathews, 1990). High levels of worry are also associated with the tendency to interpret emotionally ambiguous events as threatening (Eysenck, Mogg, May, Richards, & Mathews, 1991; Hayes, Hirsch, Krebs, & Mathews, 2010; Hirsch, Hayes, & Mathews, 2009; Mathews & MacLeod, 2005). Thus when thinking about an uncertain future event, worry-prone individuals tend to make relatively threatening interpretations and direct their attention to potential negative outcomes, whereas others are likely to interpret the same situation in a more benign manner and be less likely to attend to negative outcomes.

Evidence is accumulating that these biases have their origins in both basic biological and environmental influences. Genetic variations, such as the low expression allele of the serotonin transporter gene, are associated with emotional processing biases, which in turn mediate increased emotional vulnerability to negative life events (Beevers, Wells, Ellis, & McGeary, 2009). Traumatic experiences in adults or children can also result in negative processing biases (e.g. Lindstrom et al., 2011). In an experimental study, Fox, Zougkou, Ridgewell, and Garner (2011) have further shown that attentional biases are acquired more readily in those with the low expression allele of the serotonin transporter gene. Genetic predisposition factors and learning experiences can thus interact in the acquisition of emotional processing biases.

Emotional processing biases are usually regarded as partially automatic in that they typically operate without awareness or deliberate intent: indeed, pre-conscious attention to emotional stimuli may be a better predictor of later physiological stress reactions than self report anxiety measures (Fox, Cahill, & Zougkou, 2010). However, attention can be consciously directed as well (and emotional interpretations can be consciously endorsed or rejected; Ouimet, Gawronski, & Dozois, 2009): in the present paper we therefore consider both automated and controlled influences on worry. The processes responsible for a negative thought first intruding into awareness are assumed to be mainly non-conscious and unintentional, so they are considered here to be relatively automatic. However, once a threatening thought has entered awareness it is subject to both habitual (automatic) and intentional (controlled) processing – for example, we may try to focus on the content of a negative thought or we may try to ignore it and redirect our attention elsewhere.

### Impairment of attentional control

Another cognitive characteristic associated with worry is an impairment of the central executive function of working memory (Baddeley & Hitch, 1974), or ‘attentional control’ (Darke, 1988; MacLeod & Donnellan, 1993; Rapee, 1993). We use the term attentional control in this paper to refer to processes underlying the ability to intentionally ignore distracting information (e.g. external noises when trying to work), or shift attention from one topic to another (Miyake et al., 2000). Attentional control is distinguished from more automated capture of attention (as when we involuntarily orient to a sudden noise) by the use of deliberate conscious processes associated with the action of higher cortical structures, such as the pre-frontal and parietal cortex. Voluntary allocation of attention is sometimes referred to as “top-down control” to distinguish it from “bottom-up” or involuntary capture of attention by powerful external stimuli independently of our intentions – indeed, such capture effects can sometimes occur despite our best efforts to prevent them.

Top-down intentional control of attention has definite limits, in that we find it difficult to actively attend to more than one thing at a time, although the ability to control attention and ignore distracters varies across individuals. In their attentional control theory of anxiety, Eysenck, Derakshan, Santos, and Calvo (2007) propose that worry is responsible for taking up control capacity, thus reducing the efficiency of cognitive task performance, although this can be compensated (up to a limit) by effort. Consistent with this view, experimental findings (Hayes, Hirsch, & Mathews, 2008) have shown that – compared to thinking about other topics – worry does indeed deplete the ability to exert attentional control, particularly in pathological worriers. However, in several experimental studies, anxious individuals have been found to perform less well on tasks requiring executive control (e.g. Bishop, 2009; MacLeod & Donnellan, 1993), even when any externally imposed threat is absent. Furthermore, anxious individuals who report greater difficulties in controlling their attention on a questionnaire measure are slower to disengage their attention from threat cues (e.g. Derryberry & Reed, 2002; Lonigan & Vasey, 2009; Peers & Lawrence, 2009). Anxious individuals with pre-existing limited control resources may thus be particularly vulnerable to any further depletion of control due to worry, increasing the difficulty they experience in disengaging from negative thoughts.

In keeping with the combined cognitive biases hypothesis (Hirsch, Clark, & Mathews, 2006), we suppose that emotional processing biases and impaired attentional control combine to maintain worry. Initially, emotional processing biases increase the activation of pre-conscious threat representations, making their intrusion into awareness more likely. Negative intrusive thoughts then develop into worry episodes due to the continuing influence of habitual emotional processing biases, together with the capture of attentional control resources by threatening content. The latter effect has been revealed in studies in which tasks requiring attentional control capacity for their performance are particularly susceptible to interference from worry (Eysenck et al., 2007; Hayes et al., 2008; Leigh & Hirsch, 2011). Such capture effects may be compounded by reduced motivation to exert such control due to maladaptive beliefs about worry (Ruscio & Borkovec, 2004; Wells, 1995; Wells & Carter, 2001).

### Control of worry in non-anxious individuals

**Fig. 1:**
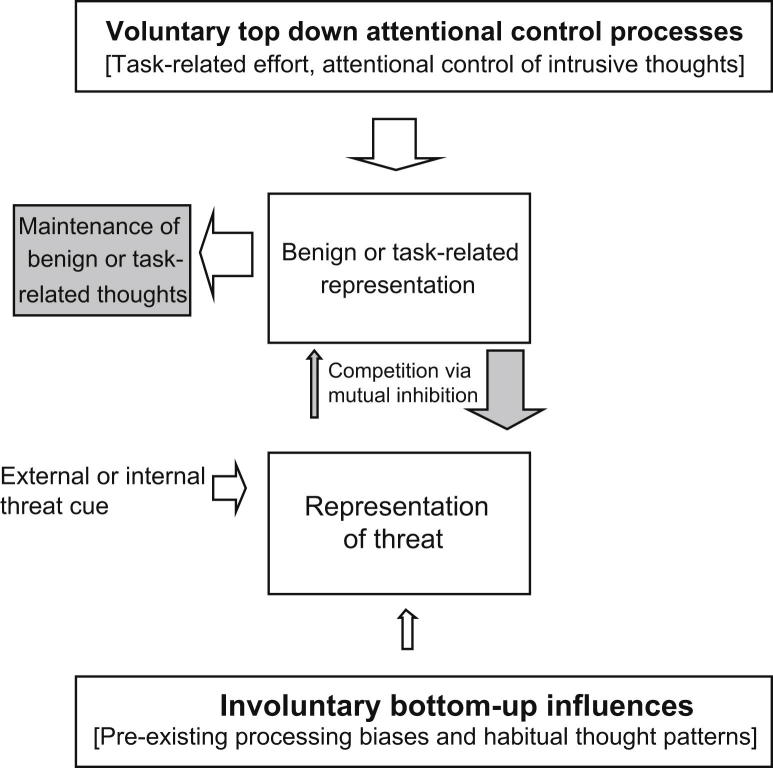
Illustration of the model as applied to a non-anxious individual able to ignore external threat cues or memories and avoid worry.

Before expanding on the processes involved in initiating and maintaining pathological worry, we first briefly illustrate how the proposed model accounts for the lack of worry in a non-anxious individual engaged in thinking about a benign topic or current task (see Fig. 1). Such mental activity requires that information about the topic or task is held as an active mental representation even if it is not currently conscious (see box labelled ‘benign or task-related representation’ in Fig. 1). Suppose that at the same time an external cue or internal reminder of some potential threat results in the formation of a competing mental representation (see box labelled ‘representation of threat’ in Fig. 1). These two representations then compete with each other for access to awareness via mutual inhibition (see filled arrows in Fig. 1), with the stronger or more active one tending to inhibit the other. Relatively stronger activation of the intended representation will thus tend to inhibit the weaker threat representation.

Each competing representation receives activation from other sources: deliberate attention activates representations of the intended task via ‘top-down control’ (or ‘concentration’); while bottom-up involuntary influences (established emotional processing biases or well-practiced negative habits of thought) activate distracting threat representations. In an individual not prone to anxiety or worry, bottom-up influences are usually too weak to activate threat representations strongly (and would be more likely to favour positive distracters). Consequently, attentional control resources deployed to keep task-related representations active are usually sufficient to inhibit competing threat representations and make it unlikely that they become dominant and intrude into awareness. Even if negative thoughts do intrude, worry can still be minimized by increasing controlled attention directed to the intended content.

### Development of worry in vulnerable individuals

**Fig. 2:**
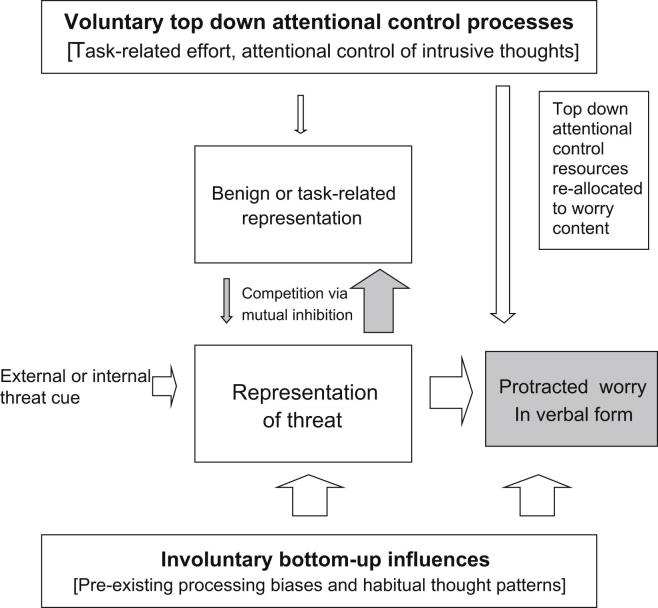
The model as applied to a worry-prone individual unable to ignore an external threat cue or memory, leading to the development of a worry episode.

Fig. 2 shows the same circumstances described above, but now for a worry-prone individual. In this case the internal representation of threat is activated more strongly than before (see larger unfilled ascending arrow in Fig. 2), due to the greater influence of involuntary bottom-up influences – emotional processing biases and well-practiced habits of thought. Consequently, the intended representation is more strongly inhibited (larger filled ascending arrow), leading to poor maintenance of attention on the intended task, while the threat representation gains strength until it intrudes into consciousness. Initially these intrusions may take different forms, including thoughts, images or impressions. However, negative intrusions in habitual worriers tend to develop into streams of related verbal thoughts about related threats (indicated by the box on the right labelled ‘protracted worry in verbal form’) which create further problems due to their tendency to persist and lead to more intrusions in the future.

In summary, negative thoughts initially intrude into awareness as the result of a combination of stronger unintentional “bottom-up” influences that serve to activate representations of threat; and insufficient voluntary “top-down” control to overcome the competing threat representations, leading to loss of attention to the original focus. Bottom-up influences include acquired processing biases and similar well-practiced habits of thought about emotional concerns. Thus the model proposes a role for past learning of processing style, so that a tendency to worry can ‘grow’ over time as processing habits become more automated with repetition and exert increasing influence over thought content. Such unintentional ‘bottom-up control’ due to prior practice is illustrated by everyday cognitive errors; such as the tendency to erroneously repeat well-practiced acts, as when we absent-mindedly take an habitual route despite originally intending to go in a different direction.

### Transition from intrusion to worry episode

Once a negative intrusion has entered awareness, additional conscious processes can come into play. Individuals not particularly prone to worry may be able to inhibit further intrusions via a modest increase in deliberate task-related effort (top-down control). In contrast, intrusions in worry-prone individuals are more likely to develop into a protracted episode of worry (see filled box on the right of Fig. 2), as a consequence of both bottom-up and top-down processes. Intrusions perceived as problems to be resolved tend to provoke efforts to deal with them taking verbal form, as if searching for possible answers to questions in the form of “What if ….?” Negative intrusions can also act as reminders of prior worry-related content and amplify habitual emotional processing biases that operate on current conscious mental content leading to escalating cycles of thought about multiple potential feared outcomes. The resulting focus on threatening topics pre-empts top-down control resources and thus makes it more difficult to interrupt the cycles of negative thought. Furthermore, motivation to re-focus attention may be undermined by beliefs that worry is uncontrollable and can even be useful, so that controlled attention will continue to be focused on worry (see right-most downward arrow in Fig. 2).

As noted above, one reason that some people become locked into cycles of worry is that habitual interpretation and attentional biases operate on the content of negative intrusive thoughts themselves (see right-most upward arrow), just as they do on pre-conscious representations of threat cues or memories. Hence the more threatening the thought content, or the more catastrophic the interpretation, the more likely it is to capture attention, promoting escalation of negative thoughts into a full-blown and protracted worry episode. Instead of this process being restrained by intentional top-down control, as in a non-vulnerable individual, attention to negative thought content takes up the very cognitive resources that would be needed to re-focus attention elsewhere.

## Experimental evidence for the assumptions of the model

### Emotional processing biases in worry-prone individuals

*Attention*: Attentional processing biases are typically revealed when task performance requires attention to non-threatening task-related content in the presence of threatening distracters – for example, when anxious individuals are required to report the *colour* of threatening words while ignoring their *meanings* in a Stroop task. Typically, pathological worry (e.g. in GAD) is associated with longer latencies to name the colours of threatening compared to healthy controls (e.g., Becker, Rinck, Margraf, & Roth, 2001; Mathews & MacLeod, 1985; Mogg, Mathews, & Weinman, 1989).

Attention to threat can be more directly assessed by presenting pairs of cues, one threatening and one non-threatening, followed by neutral targets in the prior location of one of them (for reviews see Bar-Haim, Lamy, Pergramin, Bakermans-Kranenburg, & Van Nzendoorn, 2007; Frewen, Dozois, Joanisse, & Neufeld, 2008). Faster responding to targets replacing threatening cues, indicating selective attention toward threat, occurs in individuals with pathological worry (Bradley, Mogg, White, Groom, & DeBono, 1999; MacLeod, Mathews, & Tata, 1986; Mogg, Mathews, & Eysenck, 1992). Unsurprising, sufficiently threatening information tends to capture attention universally, and differences between high and low worry groups are most apparent with moderate rather than severe threat cues (Wilson & MacLeod, 2003), indicating that pathological worriers have a lower threshold for prioritizing attention to threats that are ignored by others.

Attentional bias in pathological worry may be characterized by readiness to shift attention *towards* threat and/or with greater delay in shifting their attention *away* from threat. Research using the Posner task (e.g. Fox, Russo, Bowles, & Dutton, 2001; Yiend & Mathews, 2001), in which a single threatening or benign cue is followed by a target either in the same location as the preceding cue, or to a different location has suggested that anxiety is characterized mainly by slower disengagement of attention to threat. However others have reported evidence for selective engagement (Koster, Crombez, Verschuere, van Damme, & Wiersema, 2006), or have pointed out that what appears to be slowed disengagement may be confounded with general slowing due to emotional interference effects (Mogg, Holmes, Garner, & Bradley, 2008). Both faster engagement and slowed attentional disengagement have been found in experiments using fearful gaze as a directional cue (Fox, Mathews, Calder, & Yiend, 2007; Mathews, Fox, Yiend, & Calder, 2003). Work by Derryberry and Reed (2002) and others has further shown how attentional control interacts with these effects. For example, highly anxious individuals reporting good attentional control disengage more rapidly from threat cues than equally anxious individuals with poor control (Derryberry & Reed, 2002; Lonigan & Vasey, 2009; Peers & Lawrence, 2009).

To summarize, it appears that worry-prone individuals are more likely to engage with threat-related information and take longer to disengage from it, although both effects may be influenced to some extent by attentional control. Attentional engagement with negative thought content is likely to contribute to the *onset* of worry (see Hirsch et al., 2011) while delayed disengagement may exacerbate the difficulty of stopping a worry episode once it has begun.

Attentional bias to threat in GAD is ameliorated following successful treatment of pathological worry (Mathews, Mogg, Kentish, & Eysenck, 1995; Mogg, Bradley, Millar, & White, 1995). Conversely, engaging in worry can have the effect of inducing attention to threat: interference effects due to threat were found to be greater after a worry period that after a control period of mental arithmetic (Oathes, Squillante, Ray, & Nitschke, 2010). Evidence that attentional bias causes worry is reviewed in a later section, but the experiments discussed here are consistent with a parallel converse effect: that is, worry can amplify bottom-up processes, such as biased attention to threat (see Fig. 2).

*Interpretation*: Evidence for an association between anxiety and interpretation bias has emerged from experiments in which reading ambiguous words or texts, are followed by tasks that reveal how they have been interpreted. For example, in lexical decision tasks (deciding if a letter-string makes up a real word), anxious individuals were relatively faster than non-anxious controls to endorse ‘cancer’ (versus ‘plant’) as a word when it followed the priming word ‘growth’, indicating that the ambiguous prime had been interpreted in terms of its more threatening meaning (Richards & French, 1992). Similarly, in the context of an ambiguous description of a job interview, socially anxious individuals were relatively faster to identify words matching threatening rather than benign inferences about the outcome (e.g. ‘fail’ rather than ‘succeed’; Hirsch & Mathews, 1997; 2000).

A more general bias to interpret information as threatening has been consistently demonstrated in GAD. Mathews, Richards, and Eysenck (1989) showed that individuals with GAD wrote down more threat spellings of ambiguously threatening homophones (words that sound the same but are spelled differently – for example die/dye). In a related study, ambiguous sentences were presented to individuals with GAD and non-clinical controls and in a later recognition test those with GAD endorsed more threatening interpretations than controls (Eysenck et al., 1991).

As with attentional bias, differential interpretation biases emerge when the threatening meaning of an ambiguous event is not completely dominant, but is less apparent to everyone (Calvo & Castillo, 2001). Consequently, relative to non-worriers, pathological worriers tend to perceive more everyday (ambiguous) events as being threatening, and will be more likely to attend to such threatening meanings than to more benign alternatives.

*Effects of competition*: The tendency for those prone to pathological worry to selectively process threat-related information is modelled here as a function of competition between alternative processing options (i.e. task-related representations versus threat representations). This assumption is based on extensive neuropsychological evidence supporting the now widely accepted ‘biased competition’ model of attention (Duncan, 2006) in which neural representations within a system have been shown to compete with each other for dominance. For example, the usual neural response to a visual object is inhibited if it is presented together with another object that, when presented alone, produces a stronger response. Furthermore, the outcome of this competitive process is ‘biased’ by top-down (e.g. current goals), as well as bottom-up influences (e.g. salience, arousal; for a review see Mather & Sutherland, 2011).

Evidence of equivalent competitive effects on emotional biases includes the finding that pathological worriers are faster to detect threatening words in lexical decision tasks than non-anxious controls, but only when two competing target strings (one being a word and one not), are presented together. When only one word (or non-word letter string) was presented, no comparable differences were found between groups (Macleod & Mathews, 1991; Mogg, Mathews, Eysenck, & May, 1991). This evidence indicates that the bias to more readily identify threats is more apparent under conditions of competing processing options.

Some findings have suggested that there are circumstances under which bias can be observed even in the apparent absence of competition. When participants had to make either an emotional (good or bad?) or a semantic decision (social or physical?) about single words, evidence of differential bias was absent when emotional categorizations were predictable (i.e. when only decisions of one type occurred in a block), but was present when the decision required was unpredictable (i.e. when decision types are mixed within presentation blocks; Pury & Mineka, 2001). This suggests that, under unpredictable conditions, anxious individuals may selectively prepare to identify possible threats, thus speeding an emotional decision when one follows.

Similarly, with more sensitive methods based on a model that draws on both accuracy and speed data, some evidence has suggested that single threat words can be better detected by anxious participants (White, Ratcliff, Vasey, & McKoon, 2010). It seems likely, therefore, that threat representations are more rapidly or easily activated in worry-prone individuals, even without overt processing competition, although such effects seem to be small and thus difficult to detect. It may be that such effects still depend on the competition for processing resources that always exists in the environment (whether external or internally generated), although admittedly this assumption is difficult to quantify and test. In any event, for the purposes of the present model, we assume that the extent of competition between processing options (whether imposed by the task or self-generated) is a critically important factor influencing the strength of emotional processing biases in pathological worry.

### Modifying emotional processing biases decreases worry

The association of emotional processing biases with anxiety and pathological worry is now well established. However, the assumption that these biases have *causal* effects on anxiety – and on worry in particular – has been untested until recently. In the last decade a number of methods have been developed to experimentally modify biases of attention (Dandeneau, Baldwin, Baccus, Sakellaropoulo, & Pruessner, 2007; MacLeod, Rutherford, Campbell, Ebsworthy, & Holker, 2002; Mathews & MacLeod, 2002) and interpretation (Grey & Mathews, 2000, 2011; Mathews & Mackintosh, 2000; Murphy, Hirsch, Mathews, Smith, & Clark, 2007; Wilson, MacLeod, Mathews, & Rutherford, 2006). Use of these methods has shown that cognitive bias modification (CBM) has significant effects on later emotional reactions to a stressful experience, even 24 h later (Mackintosh, Mathews, Yiend, Ridgeway, & Cook, 2006). Within the present model, these modification or ‘training’ effects are assumed to operate primarily via bottom-up influences on acquired habits of processing threat. Emotional processing habits may be acquired naturally via incidental learning or can be induced experimentally by repeated practice in accessing threatening or non-threatening meanings (for a review, see Hertel & Mathews, 2011).

The role of attention in worry has been investigated by allocating high worriers to either experimental training designed to reduce attention to worry-related information, or a control condition not intended to modify attention (Hayes, Hirsch, & Mathews, 2010; see also Krebs et al., 2010). The training used by Hayes, Hirsch, Krebs, et al. (2010) and Hayes, Hirsch, and Mathews (2010) consisted of two consecutive tasks: the first involved pairs of one worry-related and one benign word followed by a to-be-detected neutral target that was always in the prior location of the benign word (cf. MacLeod et al., 2002); and the second involved listening to benign descriptions presented to one ear whilst ignoring worry-related content presented at the same time to the other (dichotic listening, cf. Mathews & MacLeod, 1986). Thus both tasks required attention to benign material while ignoring worry-related information, with increasing similarity to realistic worry content over tasks. The control condition used similar material but with attention being directed equally often to benign or to worry-related content. Effects were tested in a “worry task” during which participants were asked to focus on their own breathing and were interrupted at unpredictable intervals to obtain a report on current thought contents that were classified as positive, negative or neutral. The most important finding was that those trained to attend to benign material reported fewer negative thought intrusions than did those in the control condition. This finding held whether thought content was rated by participants themselves or an assessor unaware of group allocation.

These results provide direct evidence that reducing attention to worry-related threats has the effect of decreasing later negative intrusions, implying that biased attention plays a causal role in worry. Furthermore, several recent studies using multiple sessions of the attention training method developed by MacLeod et al. (2002) have found reduced worry in high worriers (Hazen, Vasey, & Schmidt, 2009) or reduced symptoms in those with GAD (Amir, Beard, Burns, & Bomyea, 2009).

Attentional bias modification techniques have also been used to investigate the relative roles of engagement and disengagement attentional processes in worry. Non-worriers were allocated to modification conditions that were designed to either modify engagement with threat/non-threat content, or disengagement from threat/non-threat content (Hirsch et al., 2011). Practice in engaging with non-threat concepts led to significantly fewer negative thought intrusions in a subsequent ‘worry task’ than did practice in engaging with threat, but disengagement conditions (threat vs. non-threat) had no differential impact on intrusions. This suggests that negative intrusions may be specifically linked to biased attentional engagement with threat; although, as discussed earlier, we suppose that disengagement processes may also be relevant to stopping worry once it has begun.

Just as attention to threatening content can increase negative intrusions, interpretation of ambiguous content in a threatening way can have similar effects. In a related experiment (Hirsch et al., 2009) high worriers were assigned either to conditions requiring benign interpretations of ambiguous words and event descriptions, or to a control condition in which interpretations were sometimes threatening and sometimes neutral. In the benign training condition, participants first resolved homographs (single ambiguous words with both threatening and non-threatening meanings such as “growth”; cf. Grey & Mathews, 2000) as cues for solving word fragments corresponding to the homographs' benign meaning (e.g. growth – pl_nt). The second task involved listening to ambiguous descriptions of worry-related events that were resolved in a benign manner (cf. Mathews & Mackintosh, 2000). Thus both tasks required the adoption of the non-threatening meanings of ambiguous material and rejection of alternatives that were made increasingly similar to realistic worry content. Those allocated to the control condition were exposed to the same material, but the ambiguity was equally often resolved in a threatening or in a non-threatening manner. The effects of training were again assessed using the assessment of the number of negative intrusions that occurred during breathing-focus periods before and after instructed worry. Compared to the control condition, fewer negative intrusions were reported by participants who had practiced exclusively benign resolutions of ambiguity, whether content was self-rated or by an independent assessor.

A further study with participants diagnosed as having GAD produced equivalent findings (Hayes, Hirsch, Krebs, et al., 2010). The benign-trained group again had fewer negative intrusions than did the control group, and a later test of emotional interpretations (Huppert, Pasupuleti, Foa, & Mathews; 2007) confirmed that they also made fewer negative interpretations. Mediation analysis demonstrated that the effect of training on negative intrusions was mediated by interpretive bias. Together, these experimental findings provide strong evidence for our assumption that emotional processing biases contribute causally to the negative intrusions that trigger worry.

### Impairment of attentional control by worry

A number of findings suggest that anxious individuals are impaired in top-down attentional control, even in the absence of experimenter-imposed threatening events. In a neuro-imaging study (Bishop, 2009), participants had to respond to a target letter while ignoring other distracting letters under conditions when targets were either easy or difficult to find. High trait-anxious individuals were slowed more by the presence of competing distracters (although group differences were apparent only when targets were relatively easy to find and so took up fewer attentional resources). This slowing was associated with less activation in dorso-lateral pre-frontal cortex, suggesting that anxious individuals recruit less effortful attentional control resources to inhibit competing distracters than non-anxious people, particularly when task demands are low (see also Fales et al., 2008). These findings are consistent with the possibility that poor attentional control is a risk factor for pathological worry, and if worry itself further impairs control, the combination will make it especially difficult to terminate worry episodes.

In a direct study of the latter possibility (Hayes et al., 2008) groups reporting high or low levels of worry were asked to worry, or think about a positive topic, while performing a random key-press task. Lapses of attention result in non-random sequences, such as pressing keys repeatedly in a fixed sequence, so that the degree of randomness achieved can be used to assess attentional control capacity assigned to the task. While worrying about their main worry topic, habitually high worriers responded less randomly in the key-press task (indicating less attentional control allocated to this task) than when thinking about their positive topic. Low worriers did not differ when thinking about positive or worry topics. However, high worriers were less random than low worriers even in the positive condition, perhaps due to trait differences in attentional control, or alternatively, because high worriers continued to worry to some extent even in the positive condition. Either way, these results provide strong support for the hypothesis that worry depletes the attentional control resources available for other tasks, and that this effect is greater in those who worry habitually.

Leigh and Hirsch (2011) replicated the above finding using a non-spatial random interval generation task and not only confirmed that worry in its usual verbal form depleted attentional control resources in high worriers, but also found that thinking about worry topics in imagery form had less effect – in this latter condition high worriers did not differ from non-worriers. Additionally the groups did not differ when performing the random interval generation task alone. These findings suggest that any trait-like impairment of attentional control in pathological worry (cf. Bishop, 2009) is exacerbated by the capture of attentional resources by worry itself. Furthermore, it seems that worry in verbal form is particularly problematic in this respect, unlike alternatives such as imagery.

As reviewed in the previous section, modifying negative interpretation bias reduces negative intrusive thoughts (Hirsch et al., 2009). More relevant in the present context, the same study also revealed that modifying negative interpretation bias reduces the depletion of attentional control resources by worry. As well as further supporting the assertion that worry depletes attentional control, these results also show that the depletion effect can be reduced by modifying processing bias and so increase ability to direct thoughts elsewhere.

### Involuntary “bottom-up” influences

Earlier models of emotional processing biases have assumed that attention to threat is promoted directly by anxiety or by hypothetical “threat evaluation systems” (e.g. Mathews & Mackintosh, 1998; Mogg & Bradley, 1998). Such models do not offer any explanation for how biases are acquired, or how they can be modified in cognitive bias modification experiments (cf. Hertel & Mathews, 2011). In the present model of pathological worry, learning effects over time contribute to ‘bottom-up’ control, by promoting the unintentional re-enactment of the same type of processing that has been practiced previously, including the processing of worry-related content.

Evidence for such unintended re-enactment effects comes from experiments in which participants practice responding to some cues (for example, names of living things), but withholding responses to others (e.g. non-living things). If response instructions are later reversed, responses are slowed to the previous “no-go” word cues in comparison to baseline, revealing that memories of previously practiced responses (or of withholding responses) were unintentionally retrieved and interfered with current goals (Verbruggen & Logan, 2008). In the same way, reminders of threats that have been repeatedly processed in particular ways in the past will tend to evoke repetition of the same type of processing, even without the deliberate intention to do so. Thus ambiguous threats that have been repeatedly interpreted previously in particular ways become more likely to be interpreted in that same way when encountered later, via bottom-up influences (e.g. Tran, Hertel, & Joormann, 2011).

### The role of verbal processing in worry

An important characteristic feature of worry is that it is predominantly verbal and relatively non-specific or general in content, as in thoughts of the type “what if the worst happens?”, in contrast to the mental images common in relaxed thought (Borkovec & Inz, 1990; ; Hoyer, Becker, & Roth, 2001). Borkovec et al. (2004) have suggested that worry reflects avoidance of images of negative outcomes, due to their greater emotional impact (although see Newman & Llera, 2011), and attempting to think about possible threats as problems to be resolved. Worry involves rather indefinite potential future dangers so that such attempts usually fail, because no satisfactory resolution exists. In experimental studies, unresolved problems are particularly likely to be remembered (the Zeigarnik effect; Zeigarnik, 1938). By analogy the unresolved threat content of worry is particularly likely to re-intrude into awareness later on.

In non-anxious groups, experiments on viewing aversive films have shown that instructions to worry afterwards lead to a greater frequency of subsequent intrusive thoughts related to the film than imagery instructions (Butler, Wells, & Dewick, 1995). As noted earlier, Leigh and Hirsch (2011) demonstrated that verbal worry had more marked deleterious effects on attentional control than imagery of worry content, but that use of imagery eliminated any differences between high and low worriers. Similarly, when high worriers were directly instructed to think about a worry topic in the form of images, rather than in their usual verbal manner, they reported fewer subsequent negative intrusions when trying to focus attention elsewhere (Hayes, Perman, Mathews, & Hirsch, 2011; Stokes & Hirsch, 2010). These findings converge on the conclusion that one (presumably unintended) consequence of verbal worry is to increase subsequent negative intrusions.

The reason for these effects of verbal worry – decreasing the ability to direct attention elsewhere and increasing later intrusions – is not entirely clear. Even if the latter effect is related to the phenomenon described by Zeigarnik (1938), this does not provide an explanation of the mechanism(s) responsible. Verbal and image representations differ in several respects: emotional images are usually concrete representations of specific outcomes, whereas verbal worry is more abstract, with the feared outcome(s) less exactly specified (Stöber & Borkovec, 2002). Consequently, pathological worriers may find it particularly difficult to dismiss negative content as unlikely or unrealistic because the negative outcomes are typically vague or unspecified, leaving them in memory as unresolved threats.

It might seem that there is a conflict between these findings and the evidence that images typically provoke more emotion than does verbal processing of the same events (e.g. Holmes & Mathews, 2010). However, when worry content is represented as an image the emotional response may be self-limiting due to the fact that imagery forces a specific concrete instantiation of previously incompletely specified worry content, so that any affective reaction can either habituate or be opposed by reappraisal of the newly specified outcome. Whatever the explanation, in several studies of high worriers instructed to imagine worry-related content, compared to worrying in their usual verbal form, the expected greater emotional response to imagery has failed to materialize (Behar & Borkovec, 2005; Hayes et al., 2011; Stokes & Hirsch, 2010). Similarly, Watkins, Baeyens, and Read (2009) found that training dysphoric individuals to ruminate in an abstract manner (that is, in similar form to verbal worry) resulted in a more negative mood than did equivalent practice in concrete thinking (which is more likely to be accompanied by imagery). Thus, although imagining a novel emotional event typically results in greater emotional reactions than does thinking about it verbally, this does not seem true in the case of worry (or rumination). In conclusion, there is little or no evidence that verbal worry serves to reduce negative emotional response, but there is a great deal of evidence that verbal worry has the undesirable effect of increasing subsequent negative intrusions.

### Beliefs and related factors serving to maintain worry

The present model is not incompatible with previous proposals that strategic process, arising from inappropriate beliefs about the advantages of worry or its corresponding dangers (Wells, 2006), or from the use of inappropriate rules for when to stop worrying (Davey, 2006), can play a part in the maintenance or development of pathological worry. Indeed, it is possible that some worry episodes are initiated voluntarily or at least are not resisted by pathological worriers. However, we assume that such strategic influences operate on worry mainly after negative thoughts have already intruded into awareness as a consequence of non-conscious underlying processes.

Furthermore, it may be that some reported beliefs about worry are *post-hoc* attempts at explaining or understanding the experience of worry, rather than being *causes* of worry (see Nisbett & Wilson, 1977; for a discussion of how post-hoc rationalizations can arise). None the less, maladaptive beliefs clearly have the potential to undermine the motivation to oppose worry via top-down effort. For example, the (negative) belief that worry is uncontrollable is likely to reduce efforts to prevent it, and the (positive) belief that worry is helpful could similarly undermine the motivation to stop worrying. Consequently, we include meta-cognitive beliefs about worry as one top-down influence in GAD, since they may divert more attentional control resources to worry content and discourage efforts to focus thoughts elsewhere.

Other researchers have developed the hypothesis that worry depends on individual differences in intolerance for uncertainty, so that those who are particularly intolerant are motivated to continue worrying in an attempt to resolve their uncertainty (Dugas et al., 1998). Support for this hypothesis comes from evidence that high worriers require more evidence in uncertain categorization tasks before arriving at a decision (Tallis, Eysenck, & Mathews, 1991). Ladouceur, Gosselin, and Dugas (2000) attempted a more direct test of the intolerance of uncertainty hypothesis by manipulating uncertainty in a gambling task and showed that this was associated with increased worry. Although these findings can be interpreted as evidence that intolerance of uncertainty causes both worry and anticipated distress in ambiguous situations, intolerance of uncertainty can also be seen as a *consequence* of biased emotional processing of threat information. To illustrate this possibility, consider a person who thinks that there is an imminent and severe threat to their future well-being (e.g. death of a loved one, financial ruin etc). Such a person is likely to exhibit intolerance of the uncertain risk and make repeated efforts to avert it, because the perceived cost of failure is so high. Evidence presented earlier suggests that emotional processing biases lead to minor or vague future threats being treated as if they were severe and costly. That is, selectively attending to the most threatening possibilities, and interpreting ambiguity in the most threatening way, is likely to result in a focus on potentially catastrophic outcomes about which any uncertainty will be perceived as intolerable.

## Further implications for the treatment of pathological worry

Evidence already reviewed has shown that modifying emotional processing biases reduces negative intrusive thoughts in vulnerable individuals (Hayes, Hirsch, & Mathews, 2010; Hayes, Hirsch, Krebs, et al., 2010; Hirsch et al., 2009). In contrast, a recent meta-analysis (Hallion & Ruscio, 2011) led to the conclusion that the overall effect of cognitive bias modification across different populations is relatively weak, although it might be useful as an adjunct treatment. However, the few studies that targeted pathological worry with multiple sessions of CBM (e.g. Amir et al., 2009) had at least moderate effect sizes, and it remains to be seen whether improved forms of CBM have even greater effects.

Existing evidence thus supports our contention that processing biases (in combination with other factors) contribute to pathological worry: none the less, our model can only be confirmed by surviving tests of newly derived predictions. We have argued that processing biases operate pre-consciously to initiate intrusions, but continue to influence conscious content and direct thinking to increasingly negative possibilities. To determine whether processing biases do indeed act on conscious content as we predict, as well as on intrusion frequency, experiments are required that investigate more directly whether training biases in a more benign direction reduces the negativity of worry content.

By contrast, we have assumed that current beliefs about worry (e.g. that worry is uncontrollable or even helpful) reduce top down attempts to control worry, but do not change the probability of negative thoughts intruding into awareness, which depend primarily on pre-conscious processes. Alternatively, it could be that beliefs can operate directly on automatic attentional processes, in the same way as do current goals (Vogt, De Houwer, Moors, van Damme, & Crombez, 2010). For example, if detecting the presence of a possible imminent danger is the current goal (rather than another task), then we would expect that top-down resources would be directed towards that goal, strengthening representations of threat cues and promoting attention to them. However, the maladaptive beliefs that have been proposed to promote pathological worry are concerned with effects attributed to worry *per se*. Consequently we assume that controlled direction of attention to worry content occurs only after a negative thought has intruded into awareness. Thus, although we predict that re-training biases should reduce both intrusion frequency and negativity of worry, we suppose that effects of modifying unhelpful beliefs about worry should be (at least initially) confined to reducing the duration of conscious worry episodes.

Note, however, that giving instructions about the contingencies involved in training can enhance the effectiveness of CBM in reducing frequency of intrusions, at least in the short term (Krebs et al., 2010). Such instructions presumably promote learning by recruiting top-down resources and directing attention appropriately; consistent with our assumption that attentional control can influence the outcome of pre-conscious competition between threatening and non-threatening representations. Thus, consciously-held goals – such as trying to maintain a specific focus during training – can influence the probability of later intrusions, although changing conscious beliefs about worry alone should not have the same effect. It remains uncertain whether explicit learning via instructions results in more durable learning over time and under conditions of stress or mental load. Further research is needed to investigate the possibility that explicit learning depends on the continuing application of attentional control resources, so that adverse conditions which reduce these resources could result in relapse. If so, then implicit learning via practice could prove more durable, because the acquired processing style should be more automated and thus less dependent on top-down attentional control.

The model we have proposed might be taken to suggest that worry would be reduced by increasing general attentional control resources. However, this prediction does not follow from the model, because the direction of any effects will depend on how such resources are deployed. We have suggested that attentional control resources in pathological worry may be pre-empted by worry and re-focused on worry content itself. Increasing control resources would thus be ineffective if attention continues to be focused on worry content, rather than on trying to switch to another topic. In any case, training intended to enhance general capacity to ignore salient cues is not always successful (Persson & Reuter-Lorenz, 2008). Rather than trying to increase general control resources, current evidence suggests that it may be more effective to train the specific control operations required such as ignoring threatening cues, in order to achieve the goal of avoiding capture of attention by worry (Schweizer, Hampshire, & Dalgliesh, 2011).

In some existing treatments for pathological worry or GAD, clients are instructed to shift attention away from worry and focus their attention on external (neutral) cues or different sensory modalities (e.g. Wells, 2006). Similarly, Borkovec and Sharpless (2006) describe ‘worry free zones’ where the client deliberately disengages from worry and focuses attention on the task at hand, as well as the use of worry timetabling in which clients try to postpone worry until a specified time period. Although these methods clearly have beneficial consequences, we suppose that these will be limited by their reliance on enhancing top-down control over worry. The ease with which processing bias can be modified has been found to predict success in the treatment of anxiety (Clarke, Chen, & Guastella, 2012) suggesting that some treatment failures may be attributable to lack of changes in these underlying processing biases. The combination of top-down and bottom-up influences in the present model leads us to the prediction that pathological worry should be most effectively treated by a combination of practice-based bias modification (to reduce negative intrusions and negativity of worry), together with interventions aimed at strengthening deliberate attempts to control and limit worry episodes.

A final therapeutic direction is suggested by the dominance of verbal linguistic processing and lack of imagery in worry (Borkovec & Inz, 1990; Hirsch, Hayes, Mathews, Perman, & Borkovec, 2012; Hoyer et al., 2001). Worriers instructed to generate imagery of worry topics, rather than worrying as usual, reported fewer negative intrusions in the following test (Stokes & Hirsch, 2010), adding to previous evidence that worry in verbal form increases later intrusions. It remains unclear why this should be the case, although we have suggested that, unlike the relatively non-specific content of worry, concrete images may promote habituation and/or their rejection as implausible. These possibilities need to be tested in future experiments, as should the therapeutic effectiveness of protracted practice in imagining worry outcomes for reducing intrusions and the negative content of worry.

## Conclusions

We have presented a cognitive model of pathological worry, focusing on the origin of intrusive negative thoughts, and the transition from intrusions to protracted worry. The central components of this model include basic processing biases in attention and interpretation that can operate independently of awareness or intent and serve to strengthen the representation of threat-related information, increasing the chance that they will break though into awareness in the form of intrusive thoughts. Such intrusions can be opposed to some extent by top-down attentional control, but once dominant, threatening thoughts in verbal form tend to pre-empt attentional control resources, making it more difficult to ignore worry-related thoughts. As well as the effects of impaired (or misdirected) attentional control, worry content continues to be influenced by acquired biases and habits of thought favouring more threatening possible meanings and outcomes. Repeated cycles of these processes can lead to worry being perceived as uncontrollable, as in GAD.

The present model borrows from several other theoretical views and incorporates the contribution of inappropriate beliefs about worry (Wells, 2006), intolerance of uncertainty (Dugas et al., 1998), and the role of verbal processing in worry (Borkovec & Inz, 1990). Despite these similarities, we suggest that prior models have not recognized the critical role of emotional processing biases, which not only serve to bring threatening thoughts into awareness, but continue to operate on the content of worry itself, augmenting and maintaining the impact of worry. We have presented evidence for these putative processes, by showing that modifying processing biases decreases the frequency of intrusive thoughts, and reduces the depletion of attentional control by worry (Hirsch et al., 2009). Other clinical researchers have gone further and shown that multiple sessions of attentional training can have a powerful effect on symptoms of GAD (e.g. Amir et al., 2009) providing additional support for the critical role of processing biases. However, the present model suggests that the combination of these methods with procedures designed to redirect attentional control and modify the usual verbal form of worry should be particularly effective. More generally, we hope that the model proposed here will further stimulate experimental and therapeutic studies designed to test and modify the various interacting cognitive influences described here.
